# AMP-activated protein kinase is involved in the activation of the Fanconi anemia/BRCA pathway in response to DNA interstrand crosslinks

**DOI:** 10.18632/oncotarget.10686

**Published:** 2016-07-18

**Authors:** Min Jeong Chun, Sunshin Kim, Soo Kyung Hwang, Bong Sub Kim, Hyoun Geun Kim, Hae In Choi, Jong Heon Kim, Sung Ho Goh, Chang-Hun Lee

**Affiliations:** ^1^ Cancer Cell and Molecular Biology Branch, Research Institute, National Cancer Center, Ilsandong-gu, Goyang, Gyeonggi, 10408, Korea; ^2^ Precision Medicine Branch, Research Institute, National Cancer Center, Ilsandong-gu, Goyang, Gyeonggi, 10408, Korea

**Keywords:** Fanconi anemia (FA)/BRCA pathway, AMP-activated protein kinase, DNA interstrand crosslinks, phosphorylation, FANCA

## Abstract

Fanconi anemia complementation group (FANC) proteins constitute the Fanconi Anemia (FA)/BRCA pathway that is activated in response to DNA interstrand crosslinks (ICLs). We previously performed yeast two-hybrid screening to identify novel FANC-interacting proteins and discovered that the alpha subunit of AMP-activated protein kinase (AMPKα1) was a candidate binding partner of the FANCG protein, which is a component of the FA nuclear core complex. We confirmed the interaction between AMPKα and both FANCG using co-immunoprecipitation experiments. Additionally, we showed that AMPKα interacted with FANCA, another component of the FA nuclear core complex. AMPKα knockdown in U2OS cells decreased FANCD2 monoubiquitination and nuclear foci formation upon mitomycin C-induced ICLs. Furthermore, AMPKα knockdown enhanced cellular sensitivity to MMC. MMC treatment resulted in an increase in AMPKα phosphorylation/activation, indicating AMPK is involved in the cellular response to ICLs. FANCA was phosphorylated by AMPK at S347 and phosphorylation increased with MMC treatment. MMC-induced FANCD2 monoubiquitination and nuclear foci formation were compromised in a U2OS cell line that stably overexpressed the S347A mutant form of FANCA compared to wild-type FANCA-overexpressing cells, indicating a requirement for FANCA phosphorylation at S347 for proper activation of the FA/BRCA pathway. Our data suggest AMPK is involved in the activation of the FA/BRCA pathway.

## INTRODUCTION

DNA interstrand crosslinks (ICLs) are a severe form of DNA damage induced by alkylating agents and platinum drugs such as cisplatin and mitomycin C (MMC) [1]. In response to ICLs, cells activate the Fanconi Anemia (FA)/BRCA pathway [2, 3]. The FA/BRCA pathway is composed of FA proteins, BRCA1/2, and other associated proteins [4]. FA is a human syndrome with diverse phenotypes including retarded growth, short stature, neurological degeneration, and a predisposition to cancer [5]. Patients with FA exhibit hypersensitivity to DNA crosslinking agents, and this observation led to the elucidation of the role of the FA/BRCA pathway in the response to ICLs [5]. FA is caused by mutations in one of 19 FA genes, which are all named with the root symbol FANC (e.g. FANCA and FANCB) [6]. The FA nuclear core complex consists of eight FA proteins including FANCA and FANCL E3 ligase, which is activated in response to DNA damage and monoubiquitinates FANCD2 [7–9]. Modified FANCD2 forms nuclear foci in regions of DNA damage and plays a role in homologous recombination repair through interaction with BRCA1 [10, 11].

FANC proteins have also been implicated in other cellular processes (reviewed in [12]). For example, FANCC and FANCA may participate in the cytokine response through interactions with signal transducer and activator of transcription 1(STAT1) and IκB kinase, respectively [13, 14]. FANCA and other FANC proteins have also been suggested to function in transcriptional regulation via interactions with Hes1 and Brg1 [15, 16]. Additionally, FANCC and FANCG are involved in the response to oxidative damage [15]. We previously demonstrated that the interaction between FANCA and the Nek2 kinase is involved in centrosome separation, and suggested a role for the FANCA protein in centrosome maintenance during cell cycle progression [17]. Similarly, other groups have described the involvement of FANC proteins in centrosome maintenance and mitotic progression [18].

To identify novel protein interactions involving FANC proteins, we previously performed yeast two-hybrid screening using FANC proteins as bait [17]. Among the candidate interacting proteins, AMP-activated protein kinase (AMPK) was selected for further analysis.

AMPK is an energy sensor that is activated in response to low ATP levels. It phosphorylates various substrates including sterol regulatory element-binding protein 1 (SREBP1), acetyl-CoA carboxylase 2 (ACC2), and tuberous sclerosis 2 (TSC2) [19, 20]. AMPK has also been implicated in the DNA damage response and control of mitotic progression [19]. In particular, it is a downstream substrate of ATM and modulates cell cycle regulators including p53 and p21 [21, 22]. Activated AMPK localizes to centrosomes during metaphase and the spindle midzone during telophase and cytokinesis [23]. Here, we determined that AMPK is required for the activation of the FA/BRCA pathway in response to DNA damage.

## RESULTS

### AMPKα interacts with FANCG and FANCA

**Figure 1:**
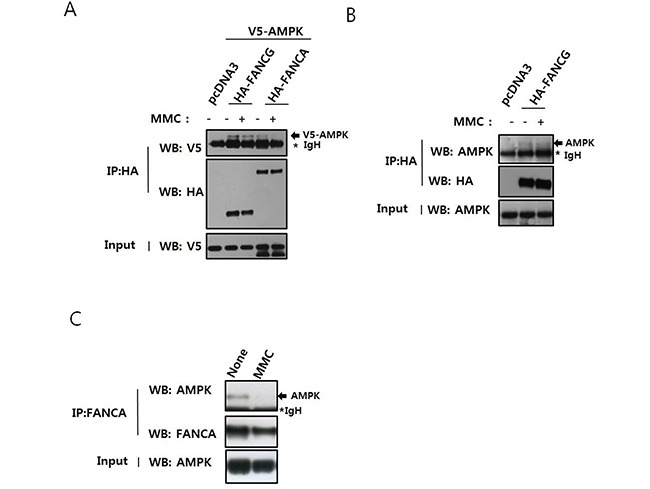
AMPK interacts with FANCA and FANCG, and this interaction is perturbed by treatment with MMC **A.** *Interaction between overexpressed V5-AMPKα1 and HA-FANCG or HA-FANCA.* HEK 293T cells were transfected with pcDNA3-V5-AMPKα1 and either pcDNA3-HA-FANCG or pcDNA2-HA-FANCA. The cells were treated with 200 ng/mL MMC for 16 h. Cell lysates were immunoprecipitated with an anti-HA antibody conjugated to agarose, and the presence of V5-AMPKα1 (indicated with an arrow) monitored by western blotting with the anti-V5 antibody (top panel). The asterisk marks the immunoglobulin heavy chain band of the antibody used for immunoprecipitation. Immunoprecipitated HA-FANCG and HA-FANCA were detected with an anti-HA antibody (middle panel). The presence of equal amounts of V5-AMPKα1 in the inputs was verified by immunoblotting the input with an anti-V5 antibody (bottom panel). **B.** *Interaction between overexpressed HA-FANCG and endogenous AMPK*α. HEK293T cells were transfected with pcDNA3-HA-FANCG and treated with MMC as in A. After immunoprecipitation with HA-agarose, endogenous AMPKα was detected by immunoblotting. **C.** *Interaction between endogenous FANCA and AMPK*α. HEK 293T cells were treated with 200 ng/mL MMC for 16 h. Cell lysates were immunoprecipitated with an anti-FANCA antibody and AMPKα detected by immunoblotting.

In a previous study, we performed yeast two-hybrid screening to analyze a novel protein network involving FANC proteins [17]. We determined that the protein kinase Nek2, which plays a role in centrosome separation, interacts with FANCA and that FANCA had a novel function at centrosomes. We also identified AMPKα1 as a FANCG-interacting protein and confirmed a direct interaction between the two proteins using pull-down assays (Supplementary Figure S1). Next, we verified the interaction in human embryonic kidney (HEK) 293T cells by co-immunoprecipitation after expression of V5-AMPKα1 and HA-FANCG (Figure 1A). In this experiment, V5-AMPKα1 was also detected in the HA-FANCA immunoprecipitates, indicating an interaction with FANCA, possibly in the FA nuclear core complex. The interactions between AMPKα1 and both FANCG and FANCA decreased after treatment with MMC. We also confirmed the interaction between HA-FANCG and endogenous AMPKα in cells transfected with pcDNA3-HA-FANCG (Figure 1B). Endogenous AMPKα and FANCA had the capacity to interact (Figure 1C), suggesting AMPK was involved in activation of the FA/BRCA pathway.

### Activation of AMPK upon induction of ICLs

**Figure 2:**
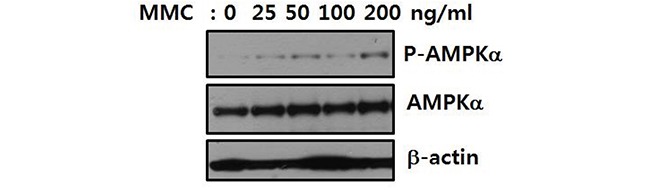
AMPK is phosphorylated upon ICL induction U2OS cells were treated with 25–200 ng/mL MMC for 8 h. Activation of AMPKα was monitored by immunoblotting with an antibody specific to AMPKα1 phosphorylated at T174 and AMPKα2 at T172 (P-AMPKα, top panel). The levels of AMPKα (middle panel) and β-actin (bottom panel) are shown as loading controls.

To analyze the involvement of AMPK in the cellular response to ICLs, we investigated whether AMPK was phosphorylated and activated by MMC treatment. Activation of AMPK was indicated by phosphorylation at T174 of AMPKα1 and T172 of AMPKα2. Treatment of U2OS cells with MMC resulted in a dose-dependent increase in phosphorylated AMPKα (Figure 2), suggesting that AMPK was involved in the cellular response to ICLs.

### AMPK knockdown inhibits MMC-induced monoubiquitination of FANCD2

**Figure 3:**
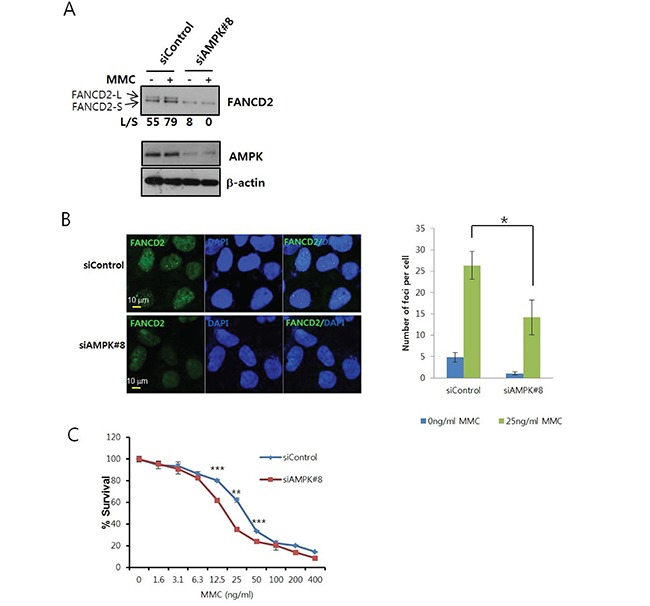
AMPK knockdown inhibits MMC-induced activation of FANCD2 **A.** *MMC-induced FANCD2 monoubiquitination is reduced in AMPK*α *siRNA-transfected U2OS cells.* U2OS cells were transfected with siRNA specific to AMPKα1 (siAMPK#8) or control siRNA (siControl). After 64 h, MMC (25 ng/mL) was added and the cells incubated for 8 h. Monoubiquitinated FANCD2 (FANCD2-L) and unmodified FANCD2 (FANCD2-S) were visualized by immunoblotting (top panel). The ratios (L/S) of band intensities of FANCD2-L and FANCD2-S are shown below the panel. Knockdown efficiency was assessed by immunoblotting with anti-AMPKα (middle panel) and anti-β-actin (bottom panel) antibodies. **B.** *Formation of FANCD2 nuclear foci is inhibited in AMPK*α *siRNA-transfected cells.* U2OS cells grown on coverslips were transfected with siAMPK#8 and then treated with MMC. FANCD2 nuclear foci (green) were visualized by immunofluorescence staining and confocal microscopy. Cells were counterstained with DAPI to stain the nuclei (blue). Representative images of MMC-treated samples are shown in the left. The number of foci per cell was counted and plotted for ≥ 13 cells (right panel). The values represent the mean ± SEM. (Student's *t*-test, *, *P* < 0.05). **C.** *Sensitization of U2OS cells following AMPK*α *knockdown*. U2OS cells were transfected with siControl or siAMPK#8 and then treated with MMC (serial two-fold dilutions from 400 ng/mL) in triplicate for 6 days. Cell viability was measured using MTT assays, and the percent survival was calculated for comparison with untreated cells. A representative graph from three independent experiments is shown. The values represent the mean ± SD. (Student's *t*-test, **, *P* < 0.01; ***, P < 0.001).

To confirm the involvement of AMPK in the activation of the FA/BRCA pathway, we assessed FANCD2 monoubiquitination and nuclear foci formation after MMC treatment in cells transfected with anti-AMPKα1 siRNA. FANCD2 monoubiquitination was readily detected by mobility shift on Tris-acetate gels. The levels of the mobility-shifted form of FANCD2 (FANCD2-L) were lower in AMPK siRNA-transfected U2OS cells (siAMPK#8) than in control siRNA-transfected cells (siControl) (Figure 3A), indicating AMPKα was required for activation of the FA/BRCA pathway. We also found that FANCD2 expression decreased after siAMPK#8 transfection. This reduction could have been caused by inhibition of FANCD2 transcription, because FANCD2 mRNA levels decreased in siAMPK#8-transfected cells in real-time quantitative reverse transcription (RT)-PCR experiments (Supplementary Figure S2). Treatment with MG132, a proteasome inhibitor, did not restore expression, indicating AMPKα did not control FANCD2 stability (data not shown). To rule out off-target effects of the siRNA, we constructed the expression vectors for siRNA-resistant AMPKα1 (pcDNA3-Res-V5-PRKAA1) by site-directed mutagenesis of pcDNA3-V5-PRKAA1. Overexpression of siRNA-resistant AMPKα1 (Res-PRKAA1) attenuated inhibition of MMC-induced FANCD2 monoubiquitination (Supplementary Figure S3A). In contrast, overexpression of the siRNA-resistant T174A mutant form of AMPKα1 did not rescue the inhibition of FANCD2 monoubiquitination (Supplementary Figure S3B). These results were confirmed in U2OS cells that stably expressed siRNA-resistant AMPKα1 using a lentiviral system (Supplementary Figure S3C). Finally, co-treatment with MMC and Compound C, a specific inhibitor of AMPK, inhibited MMC-induced FANCD2 monoubiquitination, indicating AMPK was required for FANCD2 monoubiquitination (Supplementary Figure S4).

Monoubiquitinated FANCD2 forms nuclear foci around regions of DNA damage. We evaluated FANCD2 nuclear foci formation using confocal microscopy, and found that the number of nuclear foci per cell was lower in siAMPK#8-transfected cells compared to siControl-transfected cells (Figure 3B). Furthermore, MTT assays revealed that MMC sensitivity increased in siAMPK#8-transfected cells (Figure 3C). In contrast, an increase in MMC sensitivity was not observed in cells that stably expressed siRNA-resistant AMPKα1 (Supplementary Figure S5).

### Identification of the AMPK phosphorylation site in FANCA

**Figure 4:**
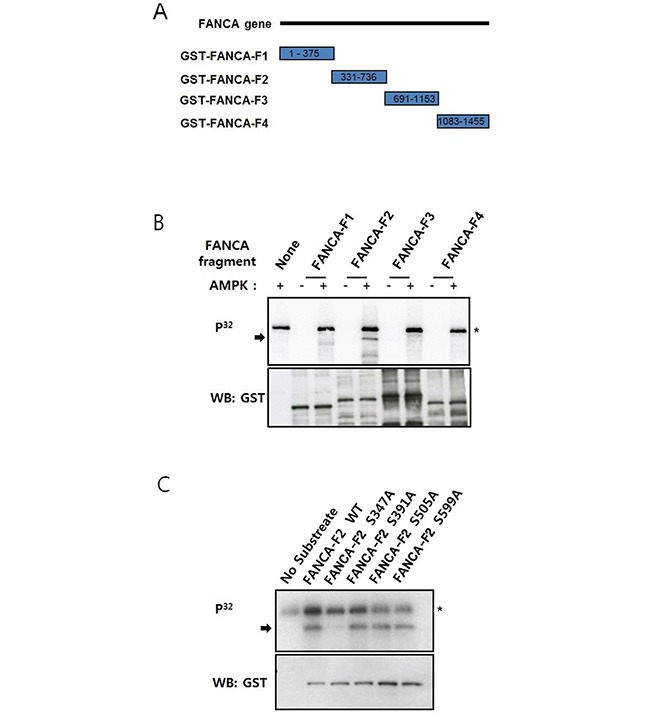
FANCA S347 is phosphorylated by AMPK *in vitro* **A.** *Schematic representation of the GST-tagged FANCA fragments used for *in vitro* kinase assays.* The numbers inside the rectangles for each fragment denote the amino acid residues in the FANCA protein sequence. **B.** *Recombinant AMPK phosphorylates a FANCA fragment comprised of amino acids 331–736.* GST-tagged FANCA fragments F1–F4 were incubated with recombinant AMPK in the presence of [γ-P32] ATP, and the reactions were subject to gel electrophoresis. Autoradiography was performed after transferring the proteins to a nitrocellulose membrane (top). Phosphorylated GST-FANCA-F2 is indicated by an arrow. The asterisk marks autophosphorylated AMPK. The substrate quantities were analyzed by immunoblotting with an anti-GST antibody (bottom). **C.** *GST-FANCA-F2 phosphorylation is abolished by the S347A mutation.* GST-FANCA-F2 fragments containing the S347A, S391A, S505A, or S599A mutations were used in the AMPK *in vitro* kinase assay as described in A.

To elucidate the mechanisms underlying AMPK-mediated activation of the FA/BRCA pathway, we tested whether AMPK could phosphorylate FA proteins. We examined FANCA phosphorylation because FANCA-defective FA-A patient fibroblasts have defects in the mitochondrial respiratory chain [24]. AMPK has also been shown to affect oxidative phosphorylation by altering the mitochondrial respiratory chain [25]. Glutathione S-transferase (GST)-fused FANCA fragments (FANCA-F1, aa #1−375 FANCA-F2, aa #331−736; FANCA-F3, aa #691−1153; FANCA-F4, aa #1083−1455) were used as substrates (Figure 4A) in *in vitro* kinase assays with recombinant AMPK. These results indicated that GST-FANCA-F2 was phosphorylated by AMPK (Figure 4B). We evaluated the sequence specificity of AMPK phosphorylation [26], mutated candidate AMPK phosphorylation sites, and then analyzed mutant GST-FANCA-F2 in *in vitro* kinase assays. These results indicated that phosphorylation was completely abolished by the S347A mutation, suggesting S347 was phosphorylated by AMPK (Figure 4C).

### Phosphorylation of S347 upon DNA damage

**Figure 5:**
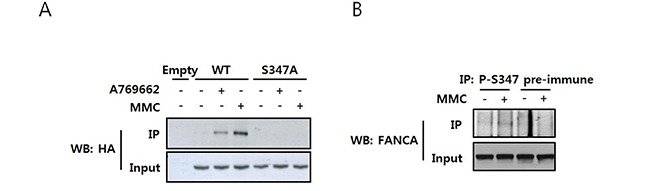
MMC induces phosphorylation of FANCA at S347 **A.** *Detection of S347-phosphorylated FANCA with a phospho-specific antibody in HA-FANCA overexpressing cells.* HEK 293T cells were transfected with pcDNA3-HA-FANCA WT or S347A and then treated with either MMC or the AMPK activator A769662 for 16 h. Cell lysates were immunoprecipitated using an antibody specific to S347-phosphorylated FANCA (P-S347) and immunoblotted with an HRP-conjugated anti-HA antibody (top panel). The presence of equal amounts of HA-FANCA in the inputs was verified by immunoblotting (bottom panel). **B.** *Detection of S347 phosphorylation of endogenous FANCA.* HEK 293T cell lysates were treated with 200 ng/mL MMC and then subjected to immunoprecipitation and immunoblotting as described in A, except S347-phosphorylated FANCA was detected using an anti-FANCA antibody (top panel).

To confirm S347 phosphorylation in cells, we generated a phospho-specific antibody against phospho-S347 (P-S347) and used it for immunoprecipitation and western blotting. The levels of P-S347 increased after MMC treatment in cells transfected with HA-FANCA (Figure 5A). In addition, the AMPK activating chemical A769662 increased P-S347 levels, suggesting S347 was phosphorylated by AMPK. In this experiment, HA-FANCA-S347A transfected samples produced no signal at all, which confirmed the specificity of the antibody. Finally, we demonstrated that phosphorylation of endogenous FANCA increased upon MMC treatment (Figure 5B).

### Overexpression of the S347A mutant abolishes MMC-induced FANCD2 monoubiquitination

**Figure 6:**
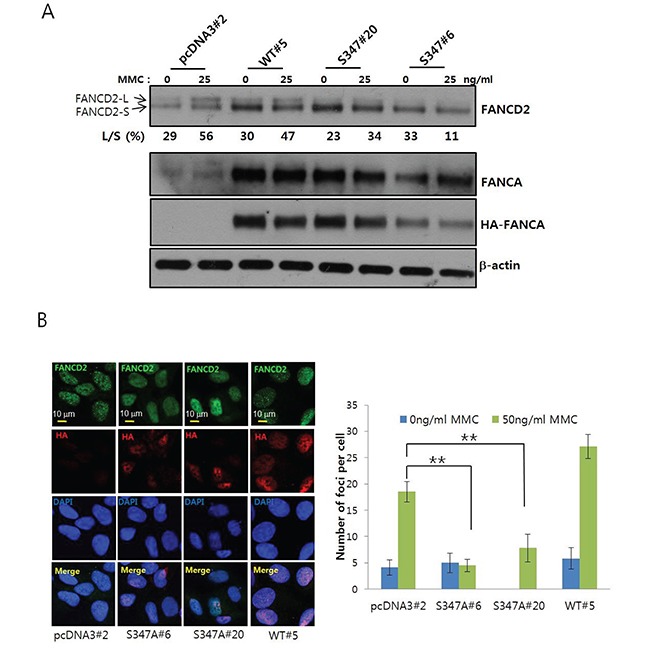
Phosphorylation of S347 may be required for proper activation of the FA/BRCA pathway upon DNA damage **A.** *Monoubiquitination of FANCD2 is inhibited in U2OS cells stably expressing the FANCA S347A mutant.* U2OS cells stably expressing HA-FANCA WT (WT#5) and the S347A mutant (S347A#6 and S347A#20) or empty vector-transfected cells (pcDNA3#2) were treated with 25 ng/mL MMC for 8 h. Monoubiquitination of FANCD2 was visualized as described in Figure 2A. **B.** *Formation of FANCD2 nuclear foci is inhibited in cells that stably overexpress FANCA S347A.* FANCD2 nuclear foci were visualized as described in Figure 2B. Representative images of MMC-treated samples are shown in the left panel. The number of foci per cell was counted and plotted (right panel). The values represent the mean ± SEM. (Student's *t*-test, **, *P* < 0.01).

To investigate the functional significance of S347 phosphorylation for activation of FANCD2, we established S347A FANCA-expressing U2OS stable cell lines (S347A#6 and #20), WT-FANCA-expressing cells (WT#5), and empty vector-transfected cells (pcDNA3#2). We then assessed MMC-induced FANCD2 monoubiquitination in these cell lines. The levels of mobility-shifted monoubiquitinated FANCD2 (FANCD2-L) were lower in S347A#20 and S347A#6 cells than in WT#5 or pcDNA3#2 cells (Figure 6A), indicating MMC-induced FANCD2 monoubiquitination was impaired in S347A-overexpressing cells. Confocal microscopy revealed a reduction in FANCD2 nuclear foci formation in S347A FANCA-expressing S347A#6 and S347A#20 cells compared to WT#5 and pcDNA3#2 cells (Figure 6B). These results suggested that S347 phosphorylation by AMPK was required for FANCD2 monoubiquitination and nuclear foci formation in response to ICLs.

## DISCUSSION

The identification of AMPKα1 as a FANCG-interacting protein prompted us to investigate a possible connection between AMPK and FA/BRCA pathway activation. An association between AMPKα and both FANCA and FANCG was detected by co-immunoprecipitation. We hypothesize that the association between AMPKα and FANCA primarily occurs at the level of the FA nuclear core complex. Co-immunoprecipitation experiments revealed a weaker interaction of HA-FANCA with V5-AMPKα1 than with HA-FANCG (Figure 1A). Additionally, we detected an interaction between AMPKα and FANCE, another component of FA core complex in co-immunoprecipitation experiments (data not shown). However, the possibility of a direct association between FANCA and AMPKα could not be completely ruled out in that a FANCA fragment (FANCA-F2) could be phosphorylated by AMPK *in vitro*. We also detected a direct association between recombinant His-tagged AMPKα1 and GST-tagged FANCA-F2 in pull-down assays (Supplementary Figure S6).

Our results indicate that AMPK, a well-known molecular sensor of energy stress, is involved in the cellular response to ICLs. AMPKα was activated by phosphorylation in response to treatment with MMC. Additionally, AMPKα1 knockdown inhibited full activation of the FA/BRCA pathway, which plays a signaling role in the cellular response to DNA damage. Finally, FANCA may be phosphorylated by AMPK at S347, which is important for activation of the FA/BRCA pathway. Consistent with these findings, a previous study described AMPK activation in response to treatment with cisplatin, which is an ICL-inducing agent [27]. AMPK is also a downstream effector of ATM, a key regulator of the DNA damage response [19, 28].

A connection between metabolic pathways and the DNA damage response is emerging. ATM, which is a key regulator in the DNA damage response, contributes to the oxidative stress response and regulates mitochondrial function [28]. In addition, metabolic pathways such as glycolysis and glutaminolysis may promote DNA damage repair in cancer cells [29]. Several transcription factors such as Myc, p53, E2F1, and E4F1 have been shown to control mitochondrial function and cell cycle checkpoints [30]. Thus, the involvement of AMPK in activation of the FA/BRCA pathway upon induction of ICLs suggests that the cellular processes that regulate the metabolic and DNA damage stress responses are closely related.

The interaction between AMPK and the FA/BRCA pathway suggests that FANC proteins might be involved in the regulation of cellular energy metabolism. Indeed, FA cells had damaged mitochondria and defects in the mitochondrial respiratory complex I [31–33]. Thus, FANC proteins may modulate cellular energy metabolism by interacting with AMPK. Notably, FANCA and activated AMPK have been reported to localize to centrosomes [17–19]. The interaction between FANCA and AMPK may also function at centrosomes and midzones during mitosis. The S347-phosphorylated form of FANCA could be enriched at these structures where it may function in the coordination of mitosis and cytokinesis.

Inhibition of AMPK with Compound C has anticancer effects in several cancer types including prostate, colorectal, and breast cancer [34–36]. Compound C also sensitizes cells to anticancer drugs such as cisplatin [37]. Given that cisplatin has DNA crosslinking activity and activates the FA/BRCA pathway, Compound C may inhibit activation of the FA/BRCA pathway. Therefore, it may be possible to overcome resistance to ICL-inducing therapies (e.g. cisplatin and MMC) by modulating AMPK activity in cancers that highly express FANC proteins in the FA/BRCA pathway [38].

Overall, our results suggest that AMPK may be required for proper activation of the FA/BRCA pathway upon DNA damage via FANCA phosphorylation. This finding may provide a rationale for exploiting AMPK as a target for anticancer chemosensitization strategies.

## MATERIALS AND METHODS

### Cells

HEK 293T cells and U2OS osteosarcoma cells were cultured in Dulbecco's Modified Eagle's medium (DMEM) supplemented with 10% fetal bovine serum (FBS; Hyclone, Logan, UT, USA) at 37°C in a humidified 5% CO_2_ atmosphere.

### Plasmids

Bait vectors for the pGBKT7-FANCA-N, pGBKT7-FANCA-M, and pGBKT7-FANCA-C fragments used in yeast two-hybrid screening have been described previously [17]. Bait vectors for full-length FANCG and pGBKT7-FANCG were constructed by inserting PCR-amplified DNA fragments into the pGBKT7 EcoRI-XhoI sites (Clontech, Mountain View, CA, USA). The primer sequences were the following: 5′-GGAATTCCATATGTCCCGCCAGACCAC-3′ and 5′-ACGCGTCGACTTTGGCAGAGATGTCCG-3′.

The pcDNA3-HA-FANCG expression vector for HA-tagged FANCG was constructed by inserting full-length FANCG cDNA into pcDNA3-HA-FANCA as described previously [17]. The pcDNA3-V5-PRKAA1 expression vector for V5-tagged AMPKα1 was constructed by inserting PCR-amplified PRKAA1 cDNA into pcDNA3-V5-Nek2 [17]. The primer sequences were the following: 5′-GGGAATTCGGCACGAGGGAAAGATG-3′ and 5′-CCCTCGAGCTGTTTATTGTGCAAGAAT-3′.

The expression vector for siRNA-resistant AMPKα1, pcDNA3-Res-V5-PRKAA1, was generated by introducing silent mutations in siRNA target sequence using oligonucleotides with the following sequences: 5’-CTCTTTCCTGAGGAcCCcTCcTAcAGTTCAACCATGATTGATG-3’ (PRKAA1_rescue#8_F) and 5’-CATCAATCATGGTTGAACTgTAgGAgGGgTCCT CAGGAAAGAG-3’ (PRKAA1_rescue#8_R). The expression plasmid for T174A AMPKα1, pcDNA3-Res-V5-PRKAA1-T174A, was produced by site-directed mutagenesis using pcDNA3-Res-V5-PRKAA1 as a template and oligonucleotides with the following sequences: 5’-GGTGAATTTTTAAGAgCAAGTTGTGGCTCACCC-3’ (PRKAA1_T174A_F) and 5’-GGGTGAGCCACAACTTGcTCTTAAAAATTCACC-3’ (PRKAA1_T174A_R). For the generation of lentiviral vectors, pLenti6-Res-PRKAA1 and pLenti6-Res-PRKAA1-T174A, pcDNA3-Res-V5-PRKAA1, and pcDNA3-Res-V5-PRKAA1-T174A were digested with EcoRI-XhoI and then subcloned into EcoRI-XhoI-CIP treated pLenti6-MCS (J.H.K personal communication), respectively.

### Co-immunoprecipitation

HEK 293T cells were transfected with pcDNA3-V5-PRKAA1 and pcDNA3-HA-FANCG or pcDNA3-HA-FANCA or the pcDNA3 empty vector using the Effectene Transfection Reagent (Qiagen, Valencia, CA, USA). The cells were treated 1 day later with 200 ng/mL MMC for 16 h and then lysed in lysis buffer (50 mM Tris-Cl, pH 7.4, 150 mM NaCl, 0.3% Igepal CA-630, 0.2% Triton X-100, 10 mM NaF, 1 mM sodium orthovanadate, and protease inhibitors). Co-immunoprecipitation was performed as described previously [17]. Briefly, cell lysates were precleared with 10 μL protein A-agarose beads (Invitrogen, Carlsbad, CA, USA) and incubated with anti-HA antibody-conjugated agarose beads (Sigma, St. Louis, MO, USA), anti-V5 antibody-conjugated agarose beads (Sigma), or an anti-FANCA antibody (A301-980A, Bethyl Laboratories, Montgomery, TX, USA) with protein A-agarose beads for 18 h at 4°C. The beads were washed with lysis buffer and subjected to sodium dodecyl sulfate-polyacrylamide gel electrophoresis (SDS-PAGE). Co-immunoprecipitated proteins were detected by immunoblotting with anti-V5 (Invitrogen), anti-FANCA (Bethyl Laboratories), anti-FANCG (Novus Biologicals, Littleton, CO, USA), or anti-AMPKα (Cell Signaling Technology, Danvers, MA, USA) antibodies.

### Transfection of siRNA

U2OS cells were transfected with synthetic siRNAs using the Lipofectamine 2000 reagent (Invitrogen) as described previously [17]. Two types of siRNAs targeting AMPKα1, Hs_PRKAA1_5 FlexiTube siRNA and Hs_PRKAA1_8 FlexiTube siRNA, were purchased from Qiagen.

### Detection of monoubiquitinated FANCD2

Lysate proteins were separated on 3–8% NuPAGE Tris-acetate gels (Invitrogen) and transferred to nitrocellulose membranes to detect monoubiquitinated FANCD2. FANCD2 was detected by immunoblotting with an anti-FANCD2 antibody (Novus Biologicals).

### Confocal microscopy

U2OS cells were grown on coverslips (18 mm diameter; Marienfeld Superior, Königshofen, Germany) in 12-well plates, fixed in 3.7% formaldehyde in phosphate-buffered saline (PBS) for 20 min, permeabilized with 0.2% Triton X-100 in PBS for 20 min, and blocked with 1% bovine serum albumin in PBST (PBS with 0.2% Tween-20). The coverslips were sequentially incubated with an anti-FANCD2 antibody (Novus Biologicals) overnight and with an Alexa 488-conjugated donkey anti-rabbit secondary antibody for 2 h. After extensive washing with PBST, the cells were counterstained with 4’, 6-diamidino-2-phenylindole, mounted on glass slides, and observed using a Zeiss Axiover LSM780 microscope and ZEN acquisition software (Carl Zeiss, Wetzlar, Germany). The number of foci per cell was counted in > 20 cells per sample. For HA-FANCA-expressing U2OS cells, we used an anti-HA primary antibody (Covance Laboratories, Dedham, MA, USA) and an Alexa 546-conjugated donkey anti-mouse secondary antibody.

### MTT assay

U2OS cells transfected with siRNA were seeded at a density of 1.5 × 10^3^ cells/well in 96-well plates and treated with MMC at the indicated concentrations. After incubation for the indicated times, each well was treated with 100 μl 0.5 mg/ml 1-(4, 5-dimethylthiazol-2-yl)-3, 5-diphenylformazan (MTT) solution in DMEM supplemented with 10% fetal bovine serum. Purple formazan crystals were allowed to develop at 37°C and dissolved in 150 μl dimethyl sulfoxide (DMSO). Absorbance at 570 nm was measured in a microplate reader (Bio-Tek, Winooski, VT, USA).

### Lentivirus production and infection

A total of 2.5 × 10^6^ 293FT cells (Life Technologies, Carlsbad, CA, USA) were plated on a 100 mm culture dish 24 h before transfection. Lentiviral construct (4.5 μg) [pLenti6-Res-PRKAA1, and pLenti6-Res-PRKAA1-T174A], 3 μg of psPAX2 (Addgene, Cambridge, MA, USA; #12260), and 1.5 μg of pMD2. G (Addgene; #12259) were co-transfected into 293FT cells using 27 μL of METAFECTENE® PRO (Biontex, Munich, Germany). The Opti-MEM® medium (Life Technologies) containing transfectant was replaced with complete medium without antibiotics 5 h after transfection. Lentivirus-containing medium was harvested 48–72 h after transfection. U2OS cells were infected with lentivirus-containing medium in the presence of 10 mg/mL polybrene (Sigma) for approximately 9–15 h and selected with blasticidin S (5 μg/mL; InvivoGen, San Diego, CA, USA).

### *In vitro* kinase assay

*In vitro* kinase assays were performed using GST-tagged FANCA fragments 1–4 (GST-FANCA-F1 to -F4), as described previously [17]. The purified GST-tagged FANCA fragments were incubated with recombinant AMPKα (72 ng, Cell Signaling #7464) in kinase buffer comprised of 60 mM HEPES (pH 7.4), 3 mM MgCl_2_, 3 mM MnCl_2_, 1.2 mM DTT, 50 μM cold ATP, and 2 μCi [γ-P^32^] ATP (Perkin Elmer, Waltham, MA, USA) at 30°C for 30 min. Samples were subjected to SDS-PAGE, transferred to nitrocellulose membranes (GE Healthcare, Milwaukee, WI, USA), and visualized by autoradiography.

### Site-specific mutagenesis

The QuikChange site-specific mutagenesis kit (Stratagene, La Jolla, CA, USA) was used to introduce alanine mutations at possible AMPK phosphorylation sites (S347A, S391A, S505A, and S599A), according to the manufacturer's protocol. The plasmids templates were pGEX-FANCA#2 or pcDNA3-HA-FANCA [17]. The oligonucleotide sequences were the following: 5′-GATGCAGAGAGAGTGGgcCTTTGCGCGGACACAC-3′ (S347A_F), 5′-GAGTGTCCGCGCAAAGgcCCACTCTCTCTGCATC-3′ (S347A_R), 5′-GGCAGAGAGTGCTCgCCTTTGTGTCTGCCC-3′ (S391A_F), 5′-GGGCAGACACAAAGGcGAGCACTCTCTGCC-3′ (S391A_R), 5′-CCGGCAAGTACCGCgCCCTCCTCACAGAC-3′ (S505A_F), 5′-GTCTGTGAGGAGGGcGCGGTACTTGCCGG-3′ (S505A_R), 5′-CCCAAAGTCCCTGACgCCCGTGTGGCGTTTATAG-3′ (S599A_F), and 5′-CTATAAACGCCACACGGGcGTCAGGGACTTTGGG-3′ (S599A_R).

### Generation of a phospho-specific antibody

Rabbits were immunized with a phospho-peptide containing the CQREWpSFART sequence.

### Immunoprecipitation and western blotting to detect phosphorylated FANCA

HEK293T cells transiently transfected with pcDNA3-HA-FANCA-WT or the S347A mutant were treated with 200 ng/mL MMC for 16 h. Cell lysates were immunoprecipitated with 5 μL phospho-specific S347 antisera (P-S347) and the immunoprecipitates were subjected to 3–8% NuPAGE Tris-acetate gel electrophoresis. FANCA was detected by immunoblotting with an HRP-conjugated anti-HA antibody (Santa Cruz Biotechnology Inc., Santa Cruz, CA, USA). The presence of equal amounts of HA-FANCA for immunoprecipitation was confirmed by immunoblotting of the lysate inputs. The P-S347 immunoprecipitates were analyzed by immunoblotting with an anti-FANCA antibody to detect endogenous S347-phosphorylated FANCA.

### Establishment of a FANCA-expressing stable cell line

U2OS cells expressing the HA-FANCA S347A mutant were established by transfecting U2OS cells with pcDNA3-HA-FANCA-WT or -S347A and selecting stable transfectants with 1 mg/mL G418 sulfate (Invitrogen). We confirmed HA-FANCA expression by immunoblotting. Positive clones were propagated in culture medium supplemented with G418 sulfate.

### Statistical analysis

The data are presented as the mean values ± SEM. Comparisons between two groups were performed using the Student's *t*-test for unpaired data.

## SUPPLEMENTARY MATERIALS FIGURES


