# Supplementary material for: AMP-activated protein kinase is involved in the activation of the Fanconi anemia/BRCA pathway in response to DNA interstrand crosslinks
Source: Oncotarget. 2016 Jul 18;7(33):53642–53. doi: 10.18632/oncotarget.10686 (PMC5288211; doi:10.18632/oncotarget.10686)
Supplement: Supplementary file 1 [file oncotarget-07-53642-s001.pdf]

AMP-activated protein kinase is involved in the activation of the Fanconi anemia/BRCA pathway in response to DNA interstrand crosslinks

SUPPLEMENTARY FIGURES

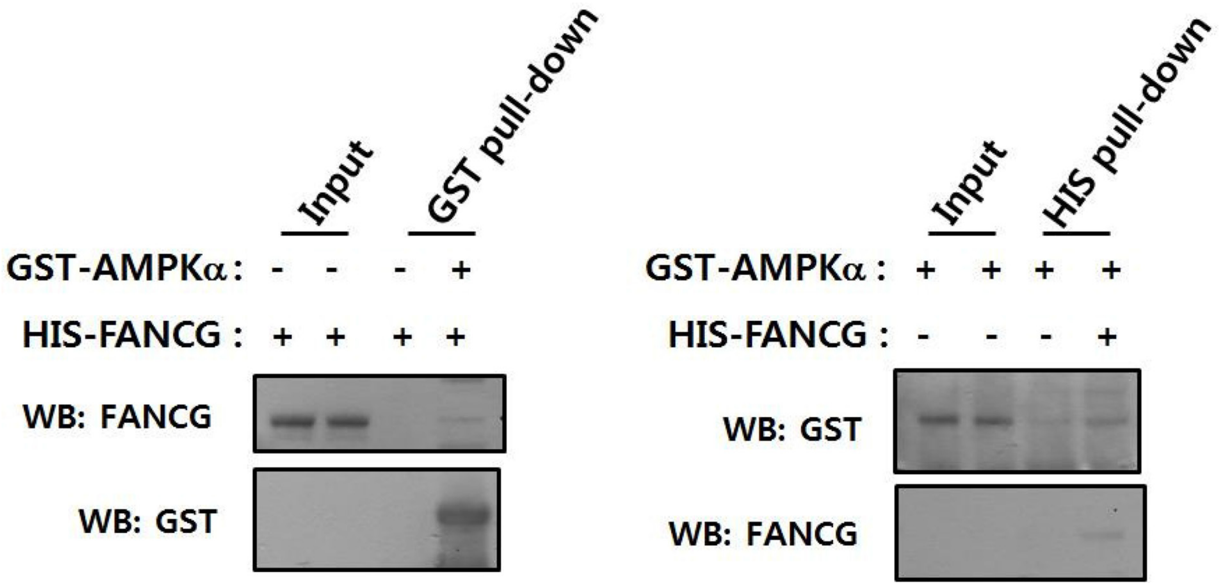

Supplementary Figure S1: HIS-FANCG and GST-AMPKα1 pull-down experiments. Theexpression vector for HIS-FANCG, pET28-FANCG, was constructed by inserting FANCG cDNA into pET28a(+) at the EcoRI-XhoI sites. The expression vector for GST-AMPKα1, pGEX-PRKAA1, was constructed by inserting PRKAA1 cDNA into pGEX5X-1 at the EcoRI-XhoI sites. Recombinant proteins were expressed in *E. coli* BL21(DE3) cells and purified through batch-wise affinity chromatography using Ni-NTA-Agarose (Thermo Fisher Scientific) or glutathione sepharose beads (Thermo Fisher Scientific). Pull-down experiments were performed using glutathione sepharose beads (right panels) and with Ni-NTA-Agarose (left panels). Bound GST-AMPKα1 and HIS-FANCG were detected with anti-GST (Santa Cruz Biotechnology) and anti-FANCG (Abnova, Taipei City, Taiwan) antibodies, respectively.

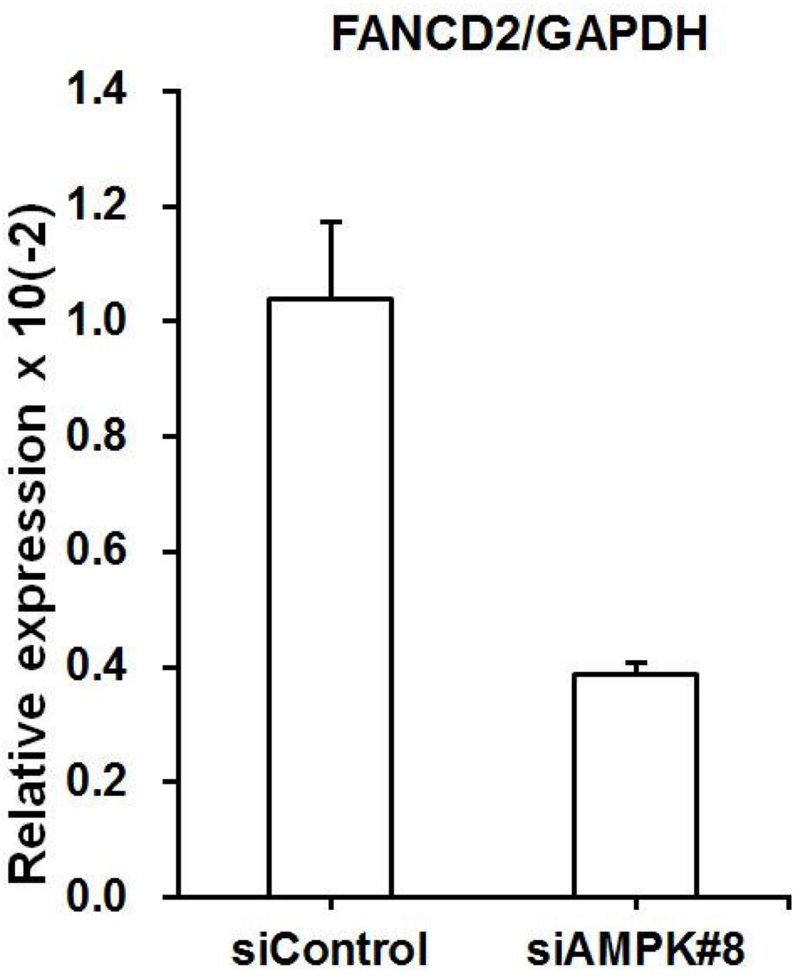

Supplementary Figure S2: AMPK knockdown decreases FANCD2 mRNA levels. Real-time quantitative RT-PCR was performed and relative expression calculated after normalizing by GAPDH mRNA levels.

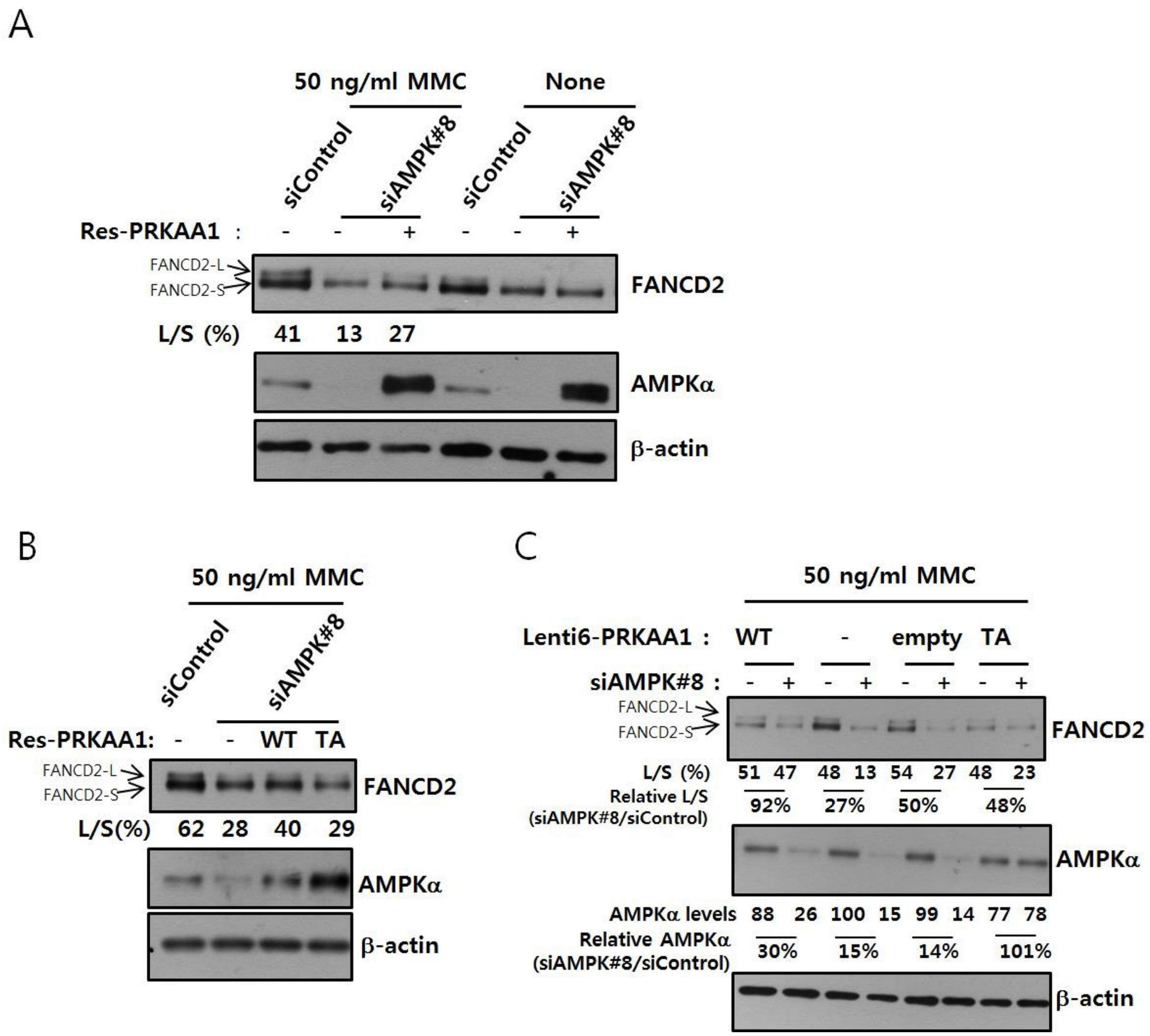

Supplementary Figure S3: Expression of siRNA-resistant AMPKα1rescues monoubiquitination of FANCD2. A. Recovery of FANCD2 monoubiquitination after expression of siAMPK#8-resistant AMPKα1 (Res-PRKAA1) with siAMPK#8 in U2OS cells. U2OS cells were cultured in 6-well plates and transfected with 80 pmol of siAMPK#8 and 2 mg of pcDNA3-Res-V5-PRKAA1 or pcDNA3 with Lipofectamine 2000. The following day, the expression plasmids were re-transfected. Three days post-transfection, the cells were treated with 50 ng/mL MMC for 8 h. FANCD2 monoubiquitination visualized as described in Figure 3A. **B.** Overexpression of the T174A mutant of Res-PRKAA1 (TA) did not rescue the inhibition of FANCD2 monoubiquitination. The experiments were performed as described in A. **C.** Monoubiquitination of FANCD2 is restored by stable expression of siRNA-resistant AMPKα. U2OS cells were infected with lentivirus harboring siRNA-resistant PRKAA1 (Lenti6-PRKAA1-WT or Lenti6-PRKAA1-T174A) or an EGFP-containing empty vector (Empty). After selection with blasticidin, the cells were transfected with siAMPK#8 or siControl and treated with 50 ng/mL MMC for 8 h.

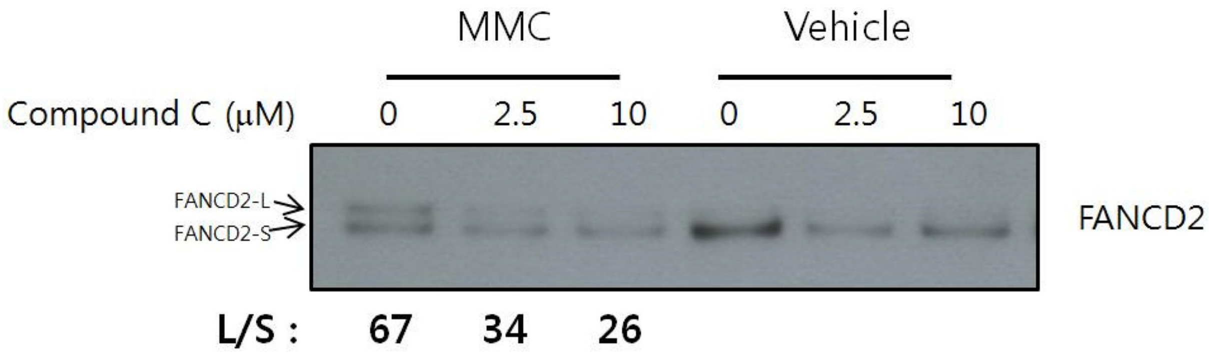

Supplementary Figure S4: Pretreatment with Compound C, a specific inhibitor of AMPK, inhibits MMC-induced monoubiquitination of FANCD2. U2OS cells were pretreated with Compound C (Merck Millipore, Darmstadt, Germany) for 1 h. MMC was then added at a concentration of 100 ng/mL. After 16 h, monoubiquitinated FANCD2 (FANCD2-L) and unmodified FANCD2 (FANCD2-S) were visualized by immunoblotting. The ratios of the levels of FANCD2-L and FANCD2-S (L/S) are shown at the bottom.

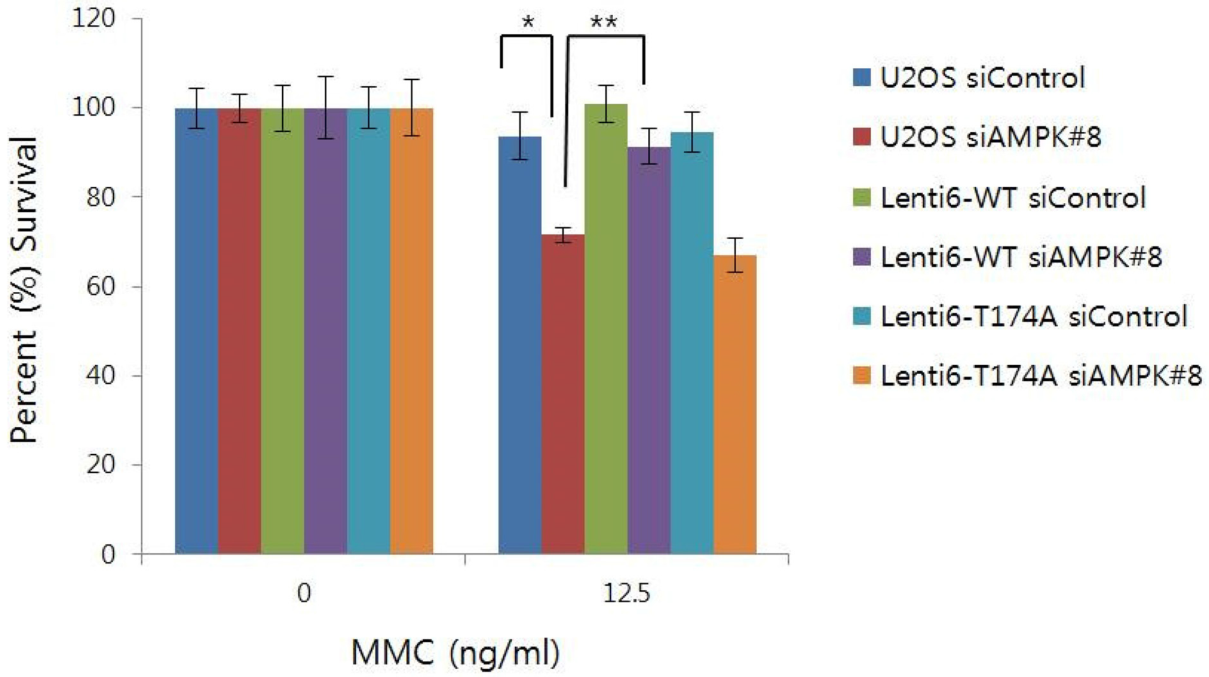

Supplementary Figure S5: Sensitization of U2OS cells after AMPKα knockdown was reversed by expression of siRNA-resistant AMPKα1. U2OS cells transduced with lentivirus harboring siRNA-resistant PRKAA1 (Lenti6-PRKAA1 and Lenti6-PRKAA1-T174A) and parental U2OS cells were transfected with siControl or siAMPK#8 and the sensitivity to MMC measured using MTT assays as described in Figure 3C. The values represent the mean ± SD. (Student's *t*-test, *, $P < 0.05$; ***, $P < 0.001$).

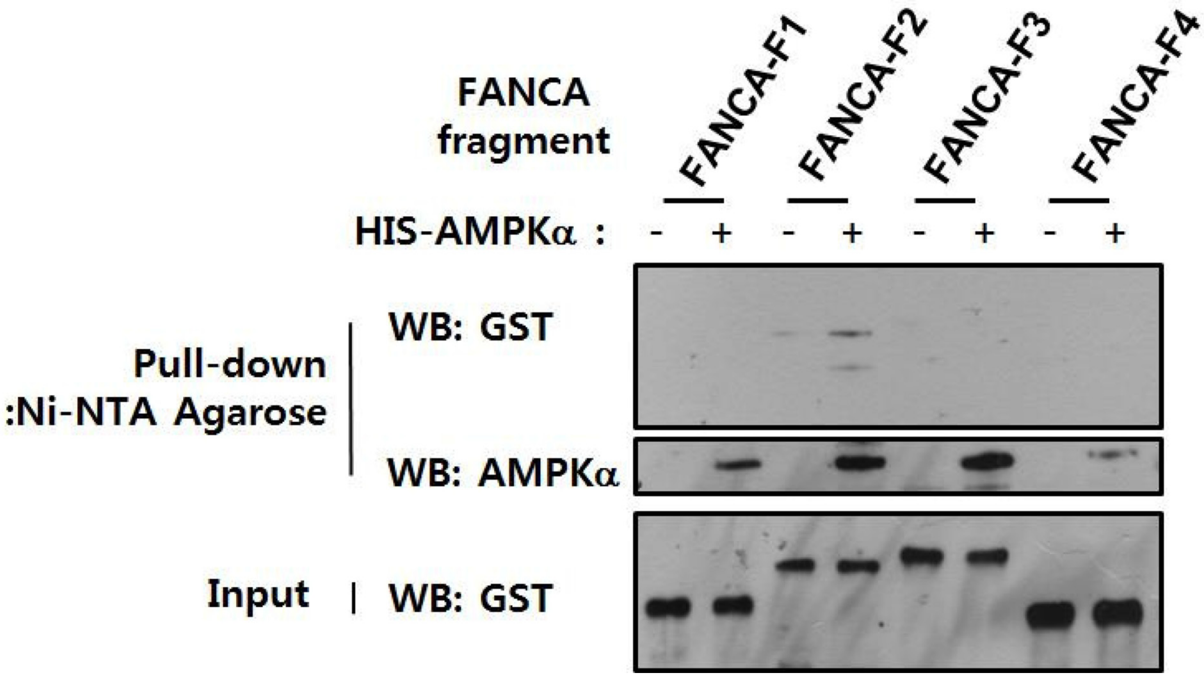

Supplementary Figure S6: Binding of the GST-FANCA fragment FANCA-F2 to HIS-AMPKα. Recombinant HIS-AMPKα purified from *E. coli* was incubated with GST-FANCA fragments (FANCA-F1, -F2, -F3, and -F4) in AMPK kinase buffer (50 mM Tris [pH 8.0] and 3 mM $MgCl_2$) for 2 h. Ni-NTA-agarose beads were then added to pull-down the recombinant HIS-AMPKα. The beads were washed three times and the presence of GST-FANCA fragments visualized by immunoblotting.
